# Physical Training and the Board of Education

**Published:** 1920-01-31

**Authors:** 


### PREVENTIVE MEDICINE AND THE GYMNASIUM.

### Physical Training and the Board of Education.

MANY times has attention been drawn in these pages to the great national benefit which is expected to arise from successful organisation of our recent maternity and child-welfare schemes and the great campaign to save the health of our under-five citizens; but the public eye is only now being attracted to many much-needed reforms in supervision of child life from this stage onwards to adolescence. To outline the many ways in which direct medical care is required from the fifth to the fourteenth year is impossible in so limited a space, and indeed, our recent articles on the functions and possibilities of the school medical services will have left a relatively clear idea of the possibilities in this direction.

### AN ALL-IMPORTANT SECTION.

One all-important section is, however, elaborated in the syllabus of physical training for schools published by the Board of Education,* which although set down primarily for guidance of the council school authorities contains suggestions of such value that they should be read by all interested, actively or otherwise, in any form of educational establishment for the young. The advice in physical training is made perhaps to apply particularly to the seven to fourteen age period, but corresponding publications have appeared for the supervision of those infants of more tender years, and from study of the whole we may derive many most useful hints for laying the ground-work of physical fitness—a prime factor in the art of preventive medicine.

### PLAY VERSUS TRAINING.

It is a common statement in many homes that little children do not need any definite physical training, since they obtain ample exercise in the ordinary movements of play. Under ideal environment this may be largely true, and also that, in their case, any developed, formal gymnastics are quite impracticable. Nevertheless under modern conditions of life, particularly in towns, there is relatively little naturally to encourage the intelligence, brightness, activity and individuality which are at once the origins and the essentials of healthy play. And even if happy spontaneous play does occur, there are "many advantages in utilising and controlling the play movements in such a way as to keep, and indeed considerably increase, their effect of happiness and enjoyment, while accustoming the boy and the mind to respond to external stimuli and suggestions, which gradually and by easy steps train the intelligence and lead up to the immediate and more accurate response, physical and mental, expected from older children."

The intention is not, however, to bring control so much as definite encouragement. Let us examine such a scheme in outline. Lessons of not more than ten to fifteen minutes should be given to little children, one in the morning and one in the afternoon. Let the morning be the primary, the all-important, and whenever possible out of doors. It should consist of simple exercises, jumping, running, dancing steps, etc., while the secondary afternoon lesson should include active games, the use of free apparatus such as balls, ropes, battledores, etc., even action-songs and singing-games. In all there must be just that amount of organisation which stimulates the child's will to take part, and if we examine the defined aims of this system, we note four cardinal points which all who control little children will do well to bear prominently in mind. As set down they are:—

### FOUR CARDINAL POINTS.

(1) To encourage alertness, independent action, and a ready response to unexpected directions.

(2) To maintain flexibility of body and so prevent or reduce the need for corrective exercises in later years.

(3) To stimulate the respiration and circulation by encouraging great activity, so aiding growth and helping to secure good health.

(4) To encourage a bright, happy, fearless independent spirit.

If all these are adequately achieved a very different type of child will in future pass on from the infant school, and one far fitter to wage the second stage of the battle for life. A not unimportant consideration is that he will, too, have learned to understand the rudiments of fair play in games. And it is not so impossible a task; instinctively the child enjoys responding to a signal, he enjoys being asked to do unexpected things quickly and to "see who can be first." If from the *outset* he is thus encouraged there never will develop that apathy which is so regrettably often seen.

### THE OLDER CHILD.

If we attempt to define the ultimate object of physical education of the older child we must survey the whole fields of the production and maintenance of health, which is here impossible even in outline, but let us remember that in the restricted circumstance of modern times it is essential that all children and young people should receive physical training by well-considered methods devised in a broad and catholic spirit to promote and encourage the health and development of mind and body. Physical education includes a host of activities likely to minister to bodily well-being, not only the gymnasium, swimming, and the natural games, but all forms of occupation which create a love of the open air and an early life of health. The syllabus before us does not pretend to deal with the subject in this wide sense, but it very definitely impresses the part, the enormously important part under modern conditions, which physical training takes.

* H.M. Stationery Office, London. Price 1s. net.

**Preventive Medicine and the Gymnasium—***(continued)*.

The early development of the infant having received due care and encouragement, we must remember that this process, in an elastic way, must be continued throughout youth. "It is essentially during the period of growth, when body, mind and character are immature and plastic, that the beneficial influence is most marked and enduring; and the highest and best results of education cannot be attained until it is realised that mental culture alone is insufficient, and that physical exercise is necessary to the development not only of the body, but also of the brain and character." The combined process must be continued through the *whole* of the growing period.

### A First Step.

As we open the pages at random, a particular drill, a particular organised game seems at first queerly uninteresting and devoid of that attractive element which has been urged above. But if, on the other hand, the sequence be considered, the careful grading of these actions noticed, and more particularly if those who will actually direct them do so in full appreciation of the spirit as well as the letter of the syllabus, then we see the whole as a foundation on which much improvement in our national physique may originate. If the first step be right and made in the right spirit, success for the revised training is assured.

"By degrees a few simple exercises may be introduced into the curriculum, which should still contain a large element of play, but play directed by the teacher. The exercises should then be gradually increased until they take the form of regular lessons on the lines indicated. It is of the greatest importance that the recreative element should never be omitted if the best results are to be gained. Enjoyment is one of the most necessary factors in nearly everything which concerns the welfare of the body, and if exercise is distasteful and wearisome its physical, as well as its mental, value is greatly diminished."

### General Principles.

Thus are the general principles wisely stated, and in the technical pages which follow the requirements have been held rigidly in mind throughout. A physical training scheme has been evolved, modified slightly, but vitally, from those of the past, which should produce an urgently needed effect, and in its present form of publication may be heartily recommended for the widest possible notice.

As with so many of those administrative changes now being instituted, the ultimate effects may not be apparent for a number of years, but, following the lessons of war-time recruiting and the amazing deficiencies in the average national physique then brought to light, it is obvious that we have to attack these problems at their root-cause and evolve schemes which, though slow in action, will be lasting in their effects. The present suggestions of the Board of Education fall aptly in with these requirements, and as such are doubly welcome.

